# Supplementary material for: TALE-based C-to-T base editor for multiple homologous genes with flexible precision
Source: Plant Biotechnol (Tokyo). 2024 Dec 25;41(4):357–65. doi: 10.5511/plantbiotechnology.24.0510a (PMC11897738; doi:10.5511/plantbiotechnology.24.0510a)
Supplement: Supplementary Data [file plantbiotechnology-41-4-24.0510a-s001.pdf]

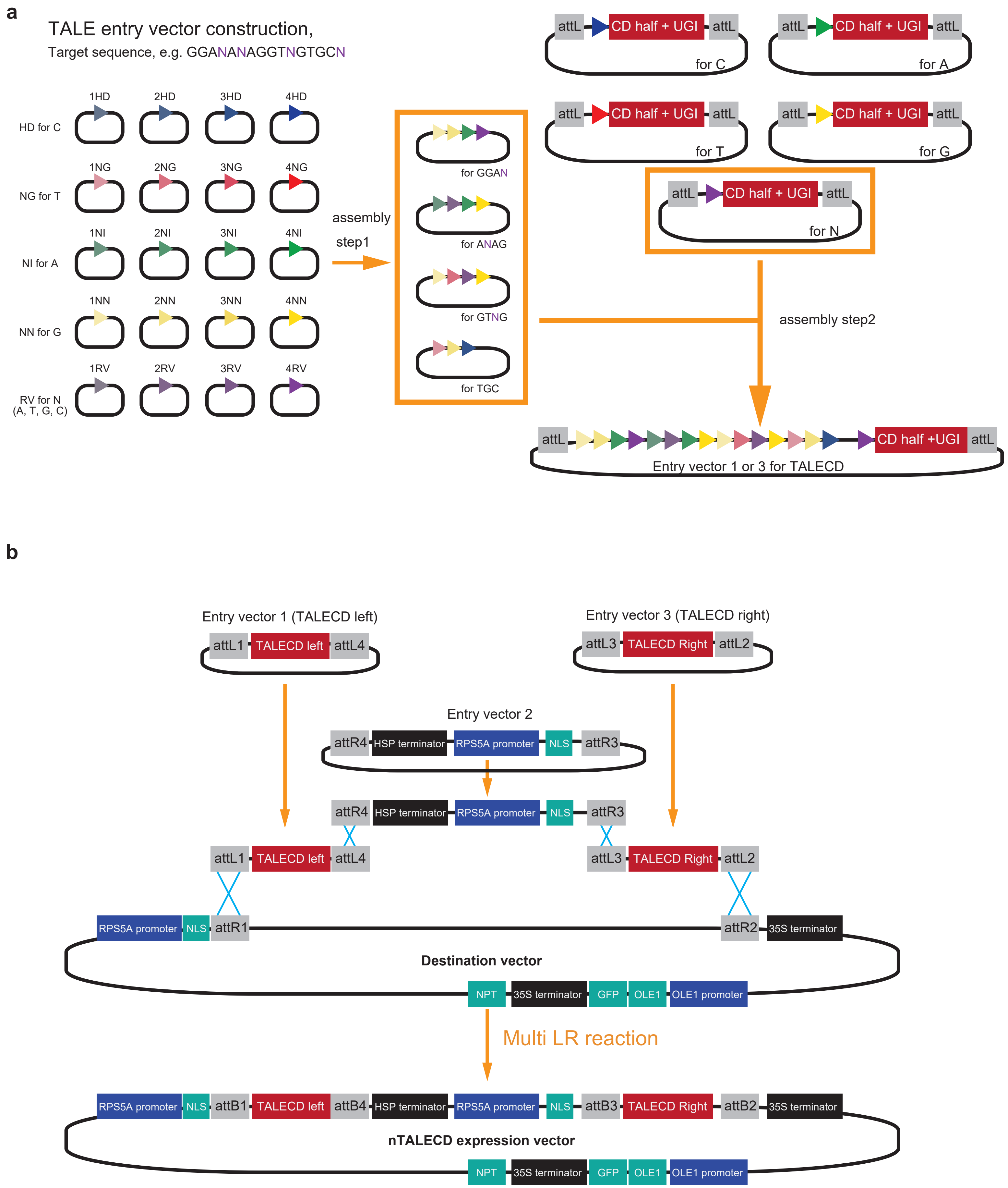

Supplementary Figure S1. 3-step nTALECD expression vector construction. (a) In assembly-step 1 and 2, entry vectors that harbor coding sequences of left or right nTALECD are assembled using each TAL-repeat module plasmid. (b) To make an entire nTALECD expression vector, multisite Gateway LR reaction (Thermo Fisher) is performed in the last step. A destination vector and an entry vector which harbor promoters, nuclear localization signals (NLS), and terminators are combined with entry vectors cloned in the former step. Details about the construction of the destination vector and entry vector 2 used in this study are in Vector construction (Materials and Methods). All component plasmids are available from Addgene. Modified from Nakazato et al., 2021.

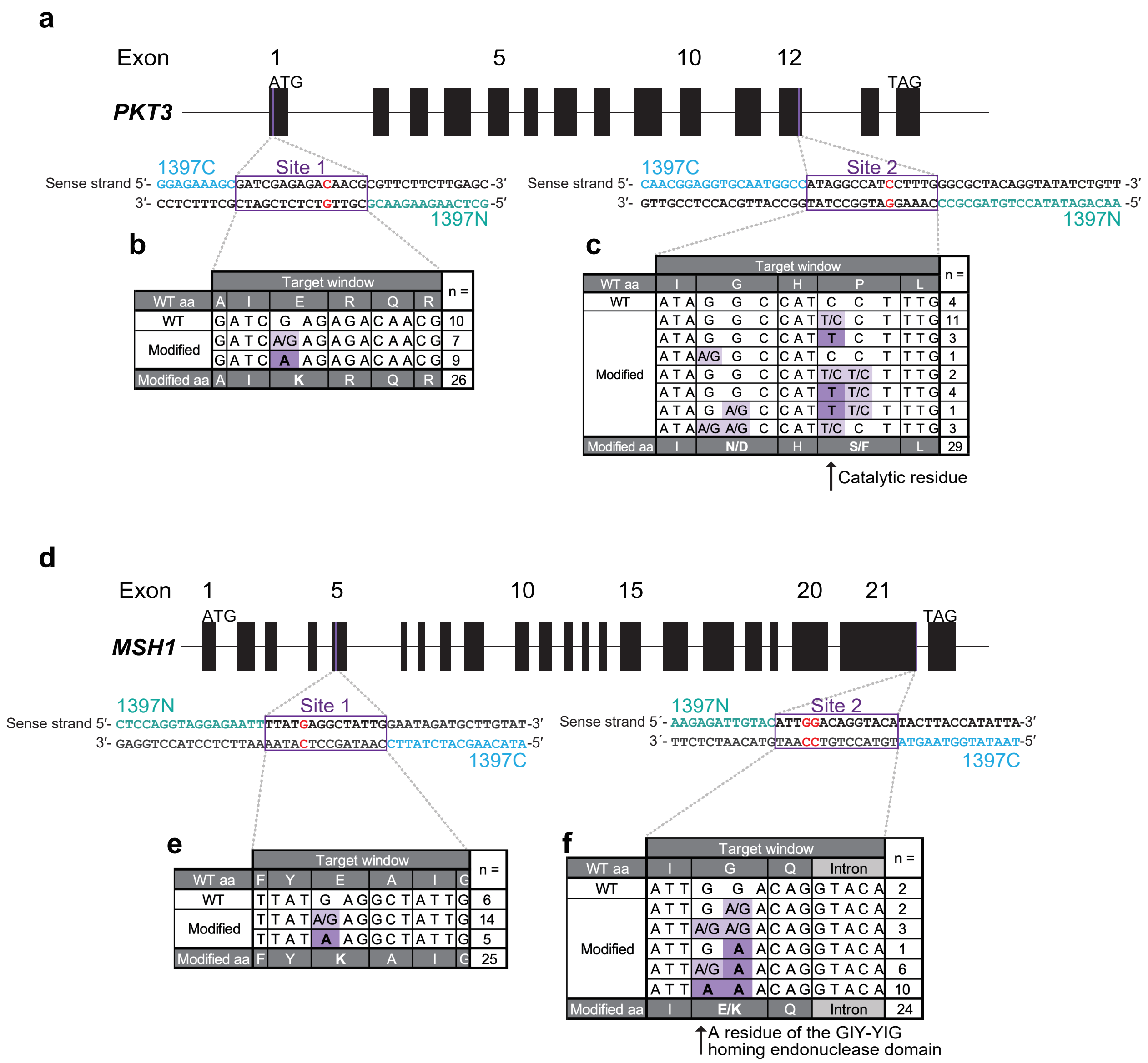

Supplementary Figure S2. Targeted cytosine base editing in *PKT3* and *MSH1* by nTALECD. (a) Genomic structure of *PKT3* and targeted sequences. Exons 1 and 12 were independently targeted by nTALECD. Green; an N-terminal half of the CD-fused TALE bind, blue; a C-terminal half of the CD-fused TALE bind. (b, c) The edited sequences in the target windows and the corresponding numbers of 21 DAS T_1 plants. *PKT3* site 1 (b) and *PKT3* site 2 (c). (d) Genomic structure of *MSH1* and targeted sequences. Exons 5 and 21 were independently targeted by nTALECD. (e, f) The edited sequences in the target windows and the corresponding numbers of 21 DAS T_1 plants. *MSH1* site 1 (e) and *MSH1* site 2 (f).

Left-1397N-Target window 1-1397C-Right

	Position in the target window															n =
	1	2	3	4	5	6	7	8	9	10	11	12	13	14	15	
WT	T	C	T	C	T	C	T	C	T	C	T	C	T	C	T	3
Modified	T	C	T	C	T	C	T	C	T	T/C	T	C	T	C	T	1
	T	C	T	C	T	C	T	C	T	**T**	T	C	T	C	T	1
	T	C	T	C	T	C	T	C	T	T/C	T	T/C	T	C	T	5
	T	C	T	C	T	C	T	C	T	**T**	T	T/C	T	C	T	5
	T	C	T	C	T	C	T	T/C	T	**T**	T	C	T	C	T	1

h/c								1		6		10				16
homo										7						

Left-1397N-Target window 2-1397C-Right

	Position in the target window															n =
	1	2	3	4	5	6	7	8	9	10	11	12	13	14	15	
WT	C	T	C	T	C	T	C	T	C	T	C	T	C	T	C	6
Modified	C	T	C	T	C	T	C	T	C	T	T/C	T	C	T	C	4
	C	T	C	T	C	T	C	T	C	T	**T**	T	C	T	C	5
	C	T	C	T	C	T	C	T	T/C	T	**T**	T	T/C	T	C	1

h/c									1		4		1			16
homo											6					

0% 100%

Right-1397N-Target window 1-1397C-Left

	Position in the target window															n =
	1	2	3	4	5	6	7	8	9	10	11	12	13	14	15	
WT	A	G	A	G	A	G	A	G	A	G	A	G	A	G	A	7
Modified	A	G	A	G	A	A/G	A	G	A	G	A	G	A	G	A	4
	A	G	A	G	A	**A**	A	G	A	G	A	G	A	G	A	1
	A	G	A	A/G	A	G	A	G	A	G	A	G	A	G	A	1
	A	G	A	A/G	A	A/G	A	G	A	G	A	G	A	G	A	2
	A	G	A	A/G	A	**A**	A	G	A	G	A	G	A	G	A	1

h/c				4		6										16
homo						2										

Right-1397N-Target window 2-1397C-Left

	Position in the target window															n =
	1	2	3	4	5	6	7	8	9	10	11	12	13	14	15	
WT	G	A	G	A	G	A	G	A	G	A	G	A	G	A	G	5
Modified	G	A	G	A	A/G	A	G	A	G	A	G	A	G	A	G	4
	G	A	G	A	G	A	A/G	A	G	A	G	A	G	A	G	2
	G	A	G	A	A/G	A	A/G	A	G	A	G	A	G	A	G	4
	G	A	A/G	A	A/G	A	A/G	A	G	A	G	A	G	A	G	1

h/c			1		9		7									16
homo																

0% 100%

Supplementary Figure S3. **The difference in conversion efficiencies among positions in 15-bp target windows.** The sequences of the target windows and those that TALEs bound to are shown in Fig. 2a. For each construct, the upper table shows allele patterns of T_1 plants at 14 DAS, and the lower table shows the positions of edited C:G pairs. In both tables, editing efficiencies are shown in tonal gradations.

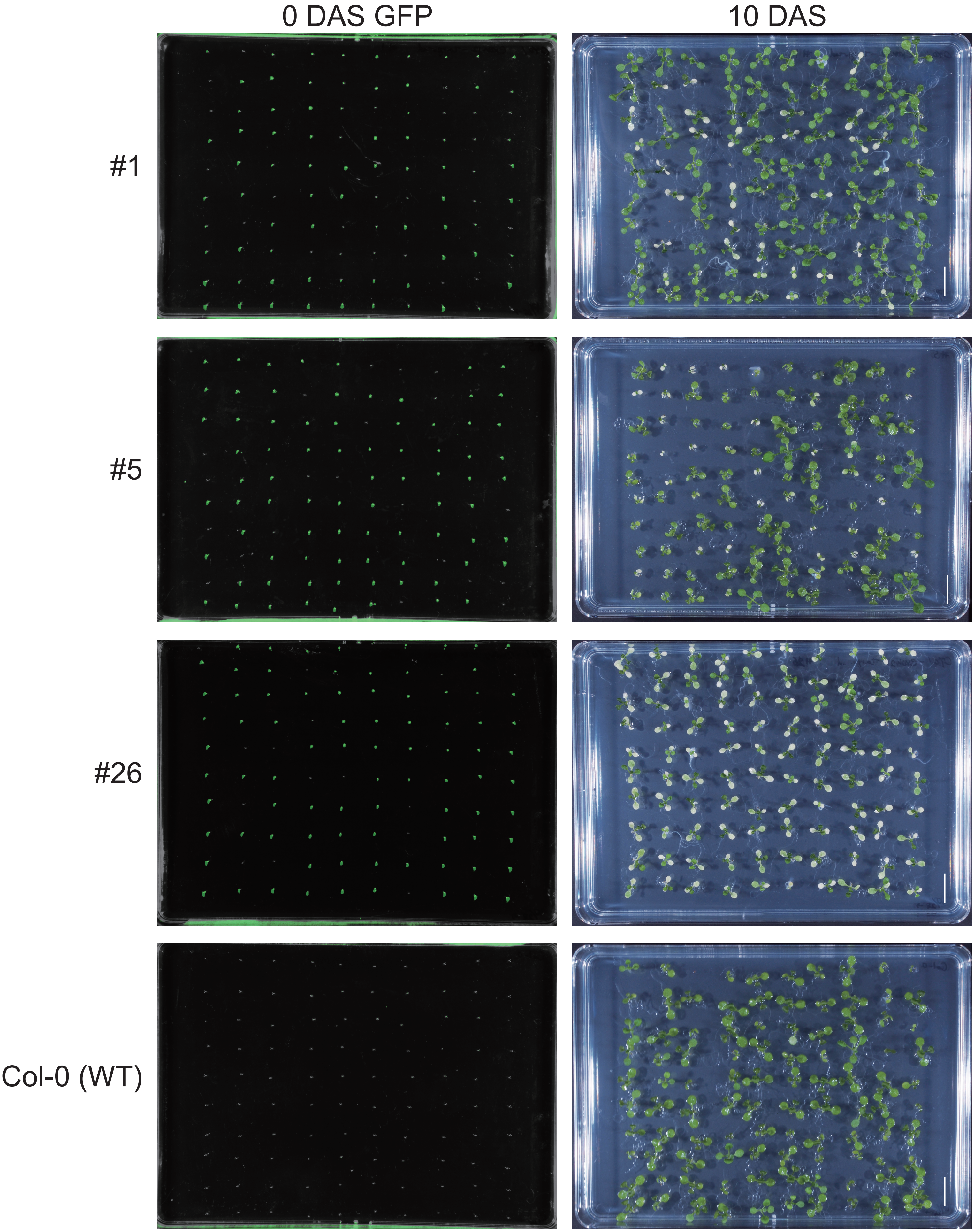

Supplementary Figure S4. Seed GFP fluorescence and 10 DAS cotyledon phenotypes of T_2 progenies of a *CYO1* site2-edited T_1 individual. The number of T_2 plants with each phenotype is shown in Fig. 1g. Scale bars, 1 cm.

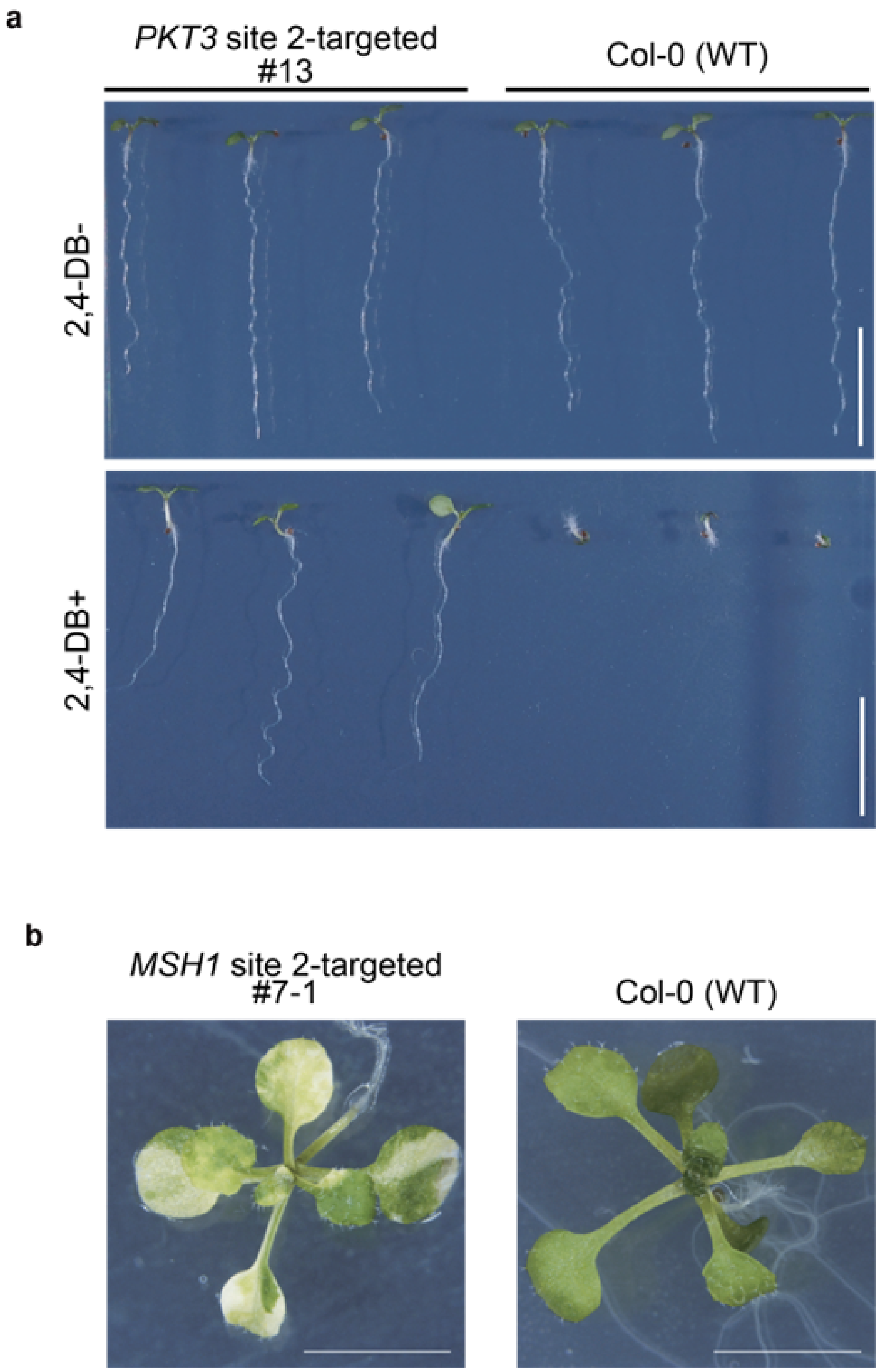

Supplementary Figure S5. **Phenotypes of base-edited T_2 plants of non-seed GFP fluorescence (null-segregants of T-DNA containing nTALECD expression cassettes).** (a) 7 DAS T_2 seedlings grown on the medium with or without 0.3 µg/mL 2,4-dichlorophenoxybutyric acid (2,4-DB). Wild-type plants were sensitive to 2,4-DB, whereas progenies of a T_1 individual whose catalytic residue of *PKT3* was edited showed resistance to the chemical, corresponding to the *pkt3* deficient mutant reported in Hayashi et al., 1998. Scale bars, 1 cm. (b) A 15 DAS T_2 seedling exhibited variegated leaf phenotype which is one of the representative phenotypes in *msh1* knockout mutant (Abdelnoor et al., 2003). Scale bars, 1 cm.

References

Abdelnoor RV, Yule R, Elo A, Christensen AC, Meyer-Gauen G, & Mackenzie SA (2003) Substoichiometric shifting in the plant mitochondrial genome is influenced by a gene homologous to MutS. Proc Natl Acad Sci. **100:** 5968–5973

Hayashi M, Toriyama K, Kondo M, Nishimura M (1998) 2,4-Dichlorophenoxybutyric acid–resistant mutants of *Arabidopsis* have defects in glyoxysomal fatty acid β-oxidation. Plant Cell **10:** 183–195

Nakazato I, Okuno M, Yamamoto H, Tamura Y, Itoh T, Shikanai T, Takanashi H, Tsutsumi N, Arimura S (2021) Targeted base editing in the plastid genome of *Arabidopsis thaliana*. Nat Plants **7:** 906–913
